# Correlations of pathomorphological parameters between lesions at the invasive front and lymph node metastases in colorectal cancer: a retrospective clinical study

**DOI:** 10.1186/s43046-024-00228-0

**Published:** 2024-07-01

**Authors:** Hui Peng, Zhifa Zhang, Yingjun Wu, Yalan Zhu

**Affiliations:** 1 Department of Pathology, The Second Affiliated Hospital of Guangzhou University of Chinese Medicine/Guangdong Provincial Hospital of Chinese Medicine, Guangzhou, 510120 China; 2 Department of Pathology, Guangdong Provincial Hospital of Chinese Medicine, Zhuhai Hospital, Zhuhai, 519000 China

**Keywords:** Colorectal cancer, Poorly-differentiated clusters, Tumor budding, Risk of lymph node metastasis

## Abstract

**Background:**

Lymph node (LN) metastasis is one of the most important indicators to evaluate stage, choose treatment strategy, and predict outcome of colorectal cancer (CRC). The morphological correlation between primary tumors and LN metastases can help predict the incidence of LN metastasis in CRC more accurately and assist with more individualized risk-stratification management decisions.

**Methods:**

A retrospective study was devised with paired tissue specimens from the invasive front of primary tumors and LN metastases in 426 patients after a radial surgery for CRC. According to the presence (N +) or absence (N-) of regional LN metastasis and the number of LN metastases (pN1a/1b/1c/2a/2b), comparisons were performed regarding tumor budding (TB) and poorly-differentiated clusters (PDC). In addition, their correlation with the incidence of LN metastasis and the extent were explored.

**Results:**

The TB and PDC in the invasive front of primary tumors presented significant correlations with the incidence of LN metastasis and the number of LN metastases in CRC (P < 0.001). TB2/3 led to a risk of LN metastasis 6.68-fold higher than TB1, while PDC2/3 resulted in a risk of LN metastasis 8.46-fold higher than PDC1. Additionally, the risk of developing 4 or more LN metastases was 3.08-fold and 2.86-fold higher upon TB2/3 and PDC2/3 than that with TB1 and PDC1, respectively. Moderate positive correlations were found between the invasive front of primary tumors and LN metastases in terms of TB and PDC, respectively.

**Conclusions:**

TB and PDC, at the invasive tumor front are important morphological markers to evaluate LN metastasis in CRC, and they can be employed as reference indicators to assess or predict the potential of LN metastasis in CRC in clinical practice.

## Introduction

Colorectal cancer (CRC) takes the third place in terms of incidence worldwide [1]. According to the Global Cancer Observatory (GLOBOCAN) 2020 database, 1,922,362 gastrointestinal cancer (GI) cancer cases were newly diagnosed and 1,497,388 deaths occurred in China in 2020, with the highest incidence in colorectal cancer (555,480 new cases; 23.90/100,000 age-standardized incidence rate) [2]. Treatment for CRC is largely dependent on the Tumor-Nodule-Metastasis (TNM) stage [3, 4]. Molecular testing and immunotherapy have developed rapidly, whereas the conventional pathomorphology review remains unsubstituted and even important in guiding treatment decisions for CRC in under-developed areas [3]. For radically resected CRC specimen, a pathology review of regional lymph node (LN) metastasis is required to guide the performance of postoperative adjuvant therapy [4]. While for pT1 malignant polyps excised endoscopically, whether an additional surgery is needed depends essentially on the risk of LN metastasis [4]. Previous research suggested that LN metastasis is under the influence of variables mainly including the depth of tumor invasion, tumor growth pattern, lymphatic invasion and extent of histological differentiation [5]. Recently, increasing studies [6–12] have found that the histological parameters at the invasive tumor front (ITF) of primary CRC tumors: tumor budding (TB) and poorly-differentiated clusters (PDC), may have correlation with the invasive capacity of tumor cells. However, few studies on the role of TB and PDC in LN metastasis have been reported yet. The present study presented the following hypothesis: TB and PDC are associated with the invasive capability of tumor cells, the occurrence of LN metastasis, and the number of LN metastases. To verify this hypothesis, paired tissue specimens from primary CRC tumors and LN metastases were collected and used for analysis.

## Methods

### Specimens collection

Human specimens were collected from patients undergoing a radical surgery for CRC between January 2020 and December 2021. From January 2020 to December 2021, the Colorectal Surgery Department of our hospital performed 2,400 surgeries, of which 445 cases (18.5%) were pathologically diagnosed as CRC. In this study, we screened samples from 426 patients (95.7%). Inclusion criteria: 1) CRC specimens confirmed by postoperative pathology; 2) simultaneous sampling of tissues from primary tumors and LN dissection; 3) absence of any anti-tumor neoadjuvant therapy before the surgery. Exclusion criteria: 1) noncurative palliative resection; 2) recurrent or metastatic lesions; 3) absence of tissue specimen from primary tumors or LN dissection; 4) the number of LN metastases < 12; 5) inability to interpret due to causes such as section loss or the section that could not be evaluated; 6) presence of other concurrent malignancies.

### Grouping method

According to the pathologic findings in the LNs, the participants were classified into two groups: LN metastasis-negative (N-) and positive (N +) groups. In the meantime, the N + group was further divided into the following 5 subgroups based on the American Joint Committee on Cancer (AJCC) staging system [3]: 1a (1 positive LN), 1b (2–3 positive LNs), 1c (0 positive LN, presence of cancerous node), 2a (4–6 positive LNs), 2b (> 6 positive LNs).

### Histopathological evaluation criteria

**Fig. 1:**
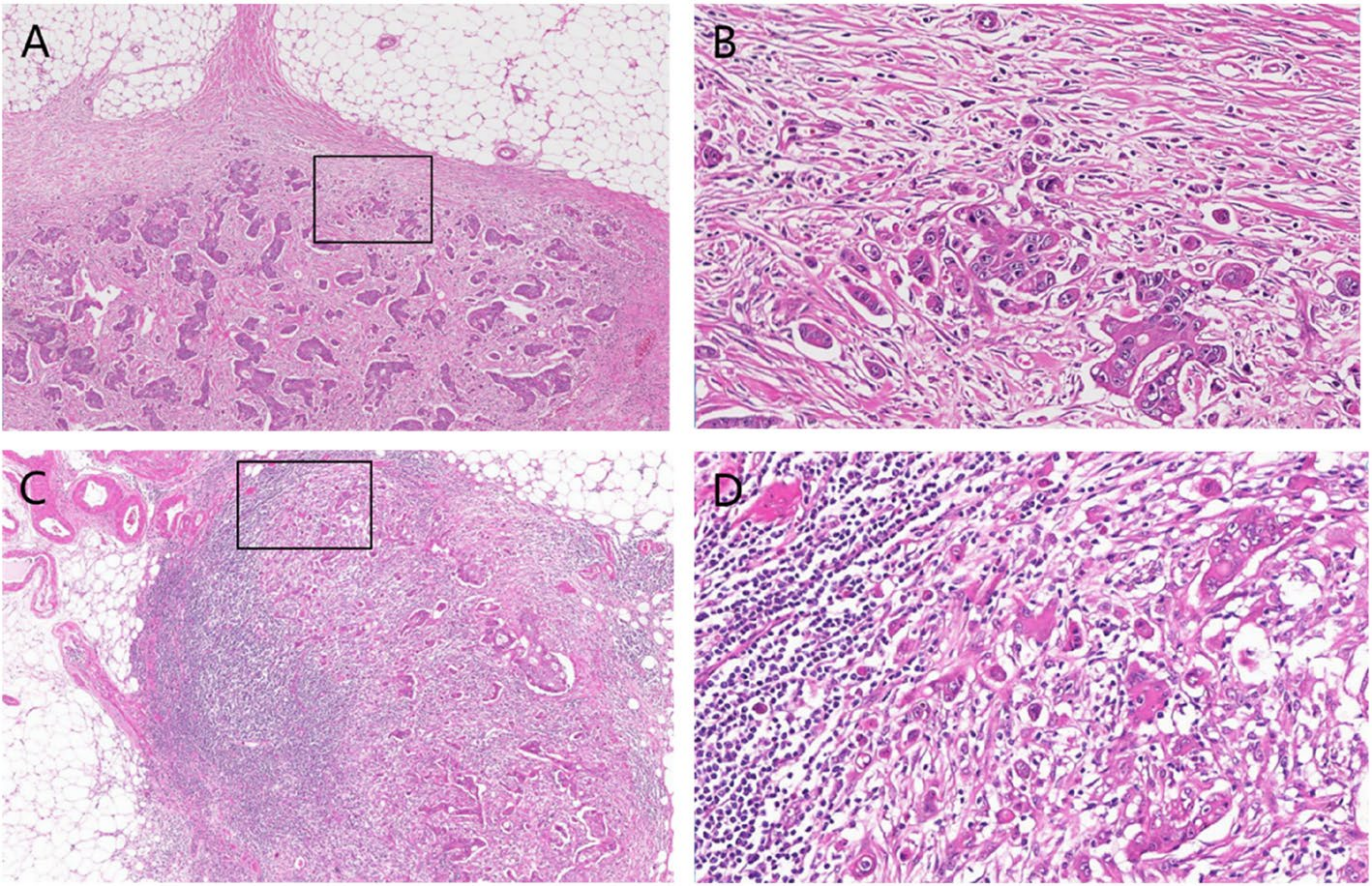
Histomorphology of Tumor Budding in the invasive front of primary tumors(**A** and **B**) and lymph node metastatic lesions(**C** and **D**) of colorectal cancer. TB were defined as a single cancer cell or a cluster of < 5 cancer cells and was graded as G1 (< 5 buds), G2 (5 to 9 buds), or G3 (≥ 10 buds) on the basis of the highest number of buds observed under an objective lens with a magnification of × 20. **A** & **B**: The same case. **B** was a partial (black box) enlargement of **A**. **C** & **D**: The same case. **D** was a partial (black box) enlargement of **C**.The magnification of **A** and **C** was × 4, the magnification of **B** and **D** was × 20

**Fig. 2:**
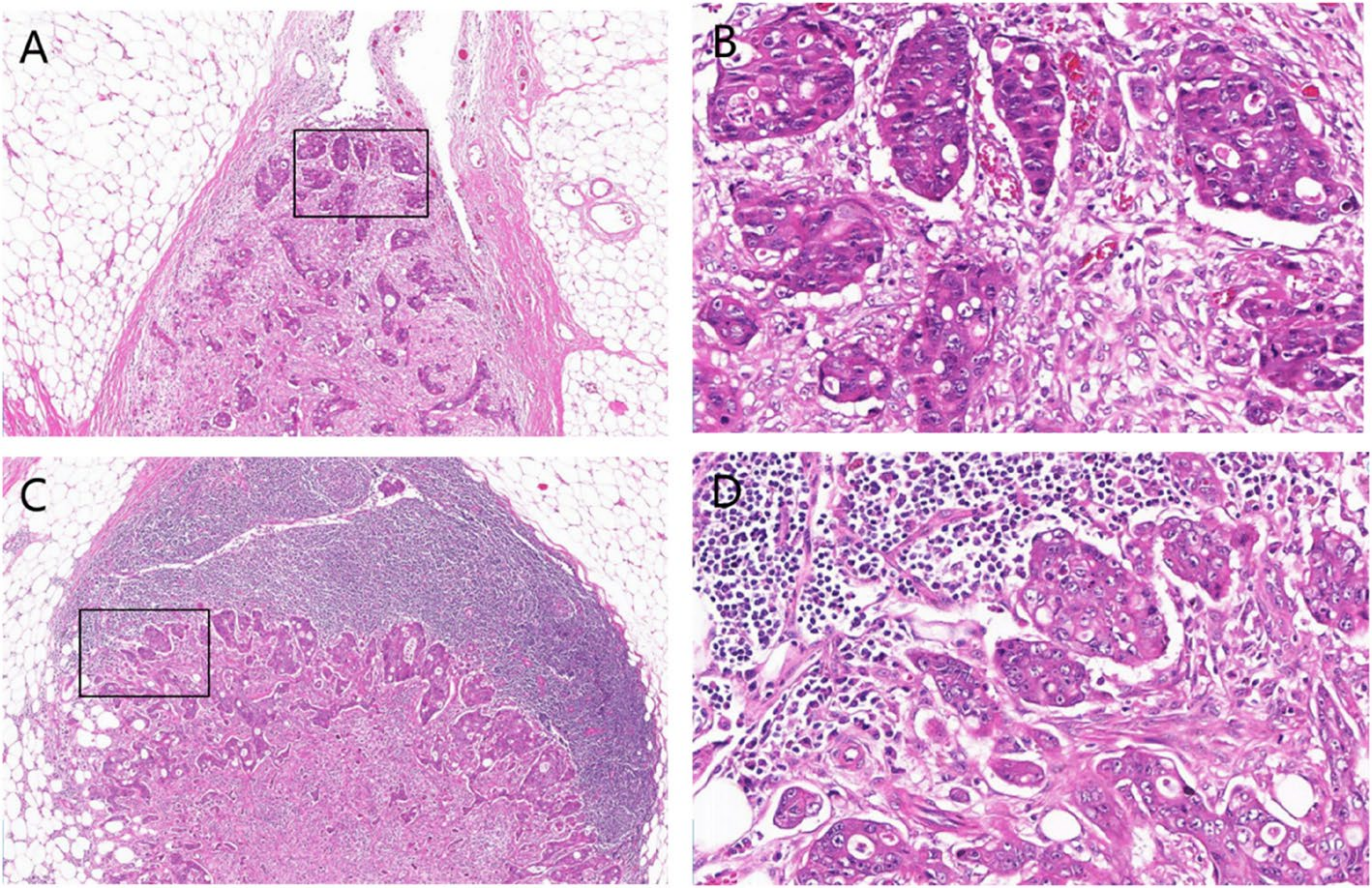
Histomorphology of Poorly differentiated clusters(PDC) in LN. PDC were defined as cancer cell clusters in the tumor stroma composed of ≥ 5 cancer cells and lacking a gland-like structure. The number of PDC in a single field of highest activity was then determined and graded as G1 (< 5 clusters), G2 (5 to 9 clusters), or G3 (≥ 10 clusters) under an objective lens with a magnification of × 20. **A** &** B**: The same case. Figure B was a partial (black box) enlargement of **A**. **C** &** D**: The same case. **D** was a partial (black box) enlargement of **C**. The magnification of **A** and** C** was × 4, the magnification of **B** and** D** was × 20

TB and PDC in primary CRC tumors and LN metastases were assessed using the same methods. TB was assessed with the following method [13]: TB was defined as a single cancer cell or a cell cluster with less than 5 cancer cells at the ITF of the Hematoxylin–eosin (HE) sections (Fig. 1), and the highest number observed microscopically (× 20 magnification) was recorded to classify the samples into 3 categories: TB1 (< 5 buds), TB2 (5–9 buds), and TB3 (≥ 10 buds). PDC was assessed based on the method of Ueno [14]: PDC was defined as cell clusters with ≥ 5 cancer cells without glandular structure at the ITF of the HE sections. All sections were first scanned at low magnification to find the hot regions at the ITF(Fig. 2), and then the number of PDC in the hot regions was counted at × 20 magnification and recorded to classify the samples into 3 categories: PDC1: < 5 clusters, PDC2: 5–9 clusters, PDC3: > 10 clusters.

### Statistical analysis

Patients’clinicopathological characteristics and outcomes are reported as numbers and percentages, mean ± SD, or median (range), where appropriate. Difference in proportion for the norminal data was assessed using the Chi-square test, Fisher’s exact test. Difference in proportion for the ordinal data was assessed using the Mann–Whitney U test or Kruskal–Wallis test. Correlation of TB/PDC grades between LN metastatic lesion and primary lesion were analyzed using Kendall’s tau (τ)-b correlation. Statistical analyses were performed using IBM SPSS Statistics for Windows, Version 25.0. (Armonk, NY: IBM Corp.) where *P* < 0.05 were considered significant.

## Results

### Patient characteristics

**Fig. 3:**
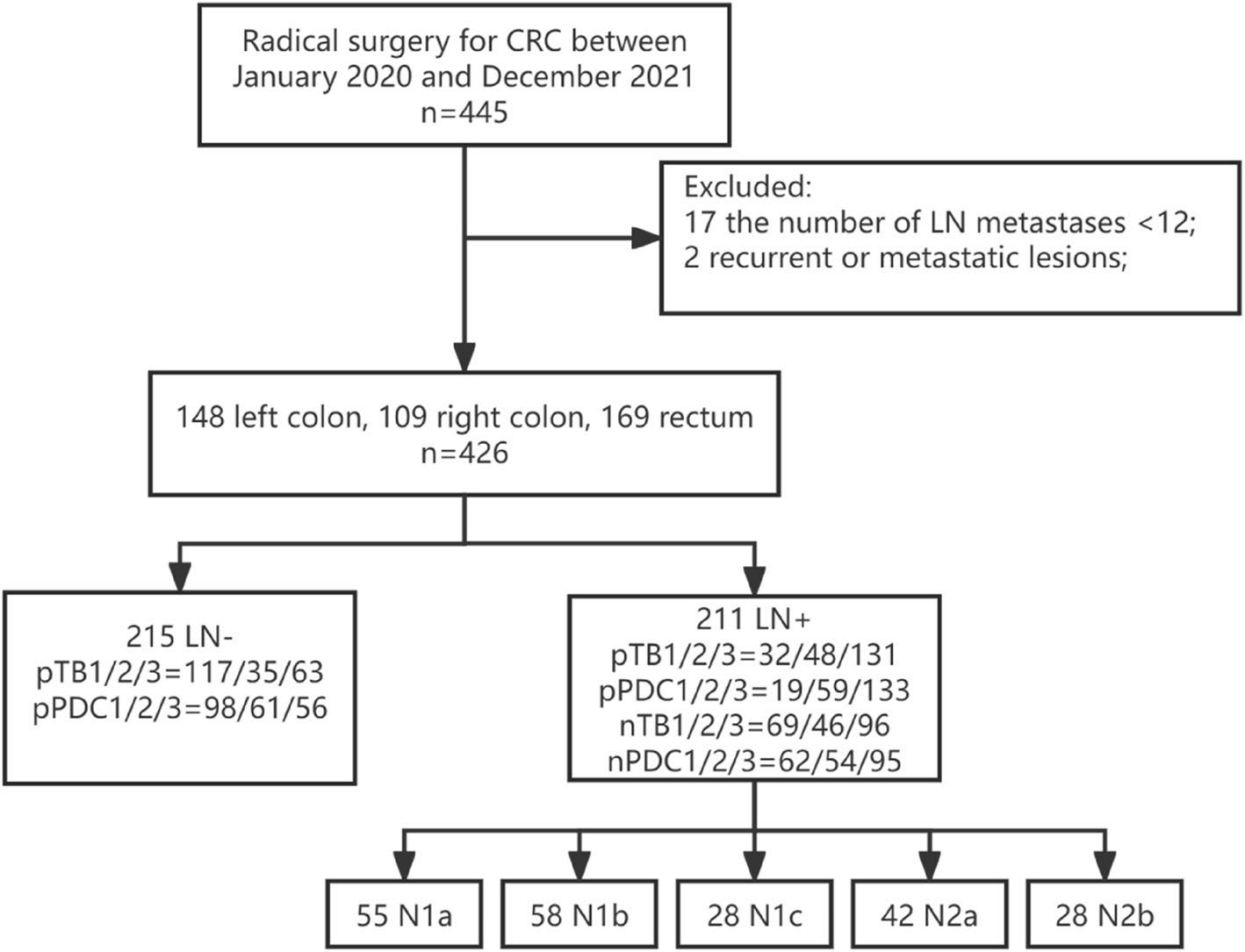
The flow diagram for this study. CRC, colorectal cancer; LN, lymph node; LN-,No lymph node metastasis;LN + ,Lymph node metastasis; pTB,Tumor budding in the invasive front of primary lesions; pPDC, Poorly differentiated clusters in the invasive front of primary lesions; nTB, Tumor budding in metastatic lesion of lymph node; nPDC, Poorly differentiated clusters in metastatic lesion of lymph node

Totally, 426 patients were enrolled, including 53.2% males (227/426), and the mean age was 64 years old (range: 31–93 years old). The mean diameter of primary tumor lesion was 4.7 cm (range: 0.9–9.0 cm). The primary tumor sites were the left colon (n = 148), the right colon (n = 109), and the rectum (n = 169). There were 211 patients in the N + group and 215 patients in the N- group (Fig. 3).

### Differential and correlation analysis of TB and PDC in primary tumors between the N- and N + groups

The proportions of TB1, TB2 and TB3 were 78.52%, 42.17% and 32.47% in the N- group while 21.48%, 57.83% and 67.53% in the N + group, and the differences between the two groups were statistically significant (Z = 8.24, *P* < 0.001). Similarly, the proportions of PDC1, PDC2 and PDC3 in the two groups were 83.76%, 50.83%, 29.63% and 16.24%, 49.17%, 70.37%, respectively, and the differences also exhibited statistical significance (Z = 9.03, *P* < 0.001) (Table 1).

**Table 1:** Difference and correlation analysis between N- /N + groups and TB/PDC grades in primary lesions of colorectal adenocarcinoma

	**TB of primary lesions**			**PDC of primary lesions**		
	**1**	**2**	**3**	**1**	**2**	**3**
**N-**	117(78.52%)	35(42.17%)	63(32.47%)	98(83.76%)	61(50.83%)	56(29.63%)
**N + **	32(21.48%)	48(57.83%)	131(67.53%)	19(16.24%)	59(49.17%)	133(70.37%)
**Z(*P*)**^**a**^	**8.24 (< 0.001)**			**9.03 (< 0.001)**		
**r(*P*)**^**b**^	**0.38 (< 0.001)**			**0.41 (< 0.001)**		

^a^Test Statistic for Mann–Whitney U test, ^b^The Correlation coefficient

Correlation analysis results demonstrated that TB in primary lesion was significantly correlated with the occurrence of LN metastasis (r = 0.38, *P* < 0.001). Comparatively, the odds ratio (OR) for TB2/3 was 6.68-fold higher than that for TB1 in LN metastasis in N + patients, suggesting a positive correlation between the TB in primary lesion and occurrence of LN metastasis. In addition, positive association was also demonstrated between the PDC in primary lesion and the occurrence of LN metastasis (r = 0.41, *P* < 0.001), and the OR for PDC2/3 in LN metastasis was 8.46-fold higher than that for PDC1 (Table 1).

### Differential and correlation analysis of TB and PDC in primary tumors between different LN + subgroups

Both TB (Z = 14.52, *P* = 0.006) and PDC (Z = 21.92, *P* < 0.001) demonstrated statistically significant differences between different N + subgroups. Additionally, TB (r = 0.14, *P* = 0.017) and PDC (r = 0.20, *P* = 0.011) also had significant correlations with different N + subgroups, respectively. Comparatively, the OR for TB2/3 and PDC2/3 in pN2 (2a, 2b) was 3.08-fold and 2.86-fold higher than that for TB1 and PDC1 (Table 2), respectively, suggesting positive correlations between TB/PDC and the number of LN metastases.

**Table 2:** Analysis of the difference and correlation between lymph node staging and TB, PDC grading in primary lesions

	**TB of primary lesions**			**PDC of primary lesions**		
	**1**	**2**	**3**	**1**	**2**	**3**
**N1a**	10 (31.25%)	15 (31.25%)	30 (22.90%)	7 (36.84%)	21 (35.59%)	27 (20.30%)
**N1b**	9 (28.13%)	13 (27.08%)	36 (27.48%)	4 (21.05%)	16 (27.12%)	38 (28.57%)
**N1c**	8 (25.00%)	7 (14.58%)	13 (9.92%)	5 (26.32%)	10 (16.95%)	13 (9.77%)
**N2a**	5 (15.63%)	10 (20.83%)	27 (20.61%)	3 (15.79%)	11 (18.64%)	28 (21.05%)
**N2b**	0 (0)	3 (6.25%)	25 (19.08%)	0 (0)	1(1.69%)	27 (20.30%)
**Z(*P*)**^**a**^	14.52 (0.006)			21.92 (< 0.001)		
**r(*P*)**^**b**^	0.14 (0.017)			0.200 (0.011)		

^a^Test Statistic for Kruskal–Wallis test

^b^The Correlation coefficient

### Differential and correlation analysis of TB and PDC in primary tumors and LN metastases

The proportions of pTB1, 2 and 3 in primary tumors were 34.78%, 31.88%, 33.33%; 13.04%, 32.61%, 54.35%; 2.08%, 11.46%, 86.46%, corresponding to nTB1, 2 and 3 in LN metastases, respectively. The differences between groups had statistical significance (Z = 54.18, *P* < 0.001) (Table 3). The proportions of PDC1, 2 and 3 in primary tumors were 24.19%, 48.39%, 27.42%; 3.77%, 41.51%, 54.72%; 1.05%, 7.37%, 91.58%, corresponding to nPDC1, 2 and 3 in LN metastases, respectively, and the differences between groups had statistical significance (Z = 70.61, *P* < 0.001) (Table 4).

**Table 3:** The difference and correlation analysis of TB grade between LN metastatic lesion and primary lesion

TB of primary lesions	TB of LN metastatic lesion		
	**1**	**2**	**3**
**1**	24 (34.78%)	6 (13.04%)	2 (2.08%)
**2**	22 (31.88%)	15 (32.61%)	11 (11.46%)
**3**	23 (33.33%)	25 (54.35%)	83 (86.46%)
**χ**^**2**^**(*P*)**^**a**^	**54.18(< 0.001)**		
**r(*P*)**^**b**^	**0.47(< 0.001)**		

^a^Test Statistic for McNemar-BowkerTest (Chi-square test),

^b^The Correlation coefficient

**Table 4:** The difference and correlation analysis of PDC grade between LN metastatic lesion and primary lesion

PDC of primary lesions	PDC of LN metastatic lesion		
	**1**	**2**	**3**
**1**	15 (24.19%)	3 (3.77%)	1 (1.05%)
**2**	30 (48.39%)	22 (41.51%)	7 (7.37%)
**3**	17 (27.42%)	29 (54.72%)	87 (91.58%)
**χ**^**2**^** (*P*)**^**a**^	70.61(< 0.001)		
**r(*P*)**^**b**^	0.54(< 0.001)		

^a^Test Statistic for McNemar-BowkerTest (Chi-square test)

^b^The Correlation coefficient

The result of correlation analysis showed a moderate correlation between the primary tumors and LN metastases in terms of TB (r = 0.47, *P* < 0.001) (Table 3) and PDC (r = 0.54, *P* < 0.001) (Table 4).

## Discussion

In this practical retrospective study, the pathomorphological parameters that affect LN metastasis in the invasive front of CRC was discussed. The innovation of this article is that the TB and PDC grading systems were used simultaneously to assess primary tumors and LN metastases. To our knowledge, the present study is the first study of LN metastasis by TB and PDC grading systems using the same standard method in China. There are two hypotheses that are validated by the results of the study: 1) high-grade TB and PDC in primary tumor are associated with a high incidence of LN metastasis; 2) the numbers of TB and PDC in primary tumors are positively associated with the numbers of TB and PDC in LN metastases.

The AJCC staging system that uses the depth of tumor invasion, LN metastasis and distant metastasis to stage tumor is regarded as a major prognostic factor indicating the clinical course of CRC [3]. Accurate staging and risk assessment for LN metastasis has significant implications for guiding treatment decisions and improving survival rate [4]. For instance, in cases undergoing radical CRC resection and regional LN dissection, it is important to perform risk assessment for LN metastasis by postoperative pathology review of the infiltration of muscularis propria, distant metastasis and other risk factors [3]. Absence of LN metastasis is classified as pT2N0M0 (clinical stage I), and the tumor can be cured by en bloc resection, with subsequent follow-up; while in the presence of 1 LN metastasis, pT2N1aM0 (clinical stage IIIa) is classified [3, 15], and postoperative adjuvant chemotherapy is required [4, 16]. Regarding the malignant rectal polyps collected endoscopically, postoperative follow-up is needed only if there is no unfavorable histology[3]; otherwise (e.g., presence of vascular or lymphatic infiltration), a second surgery may be warranted due to an increased risk of LN metastasis [16, 17]. Usually, LN metastasis is associated with the factors including vascular invasion and the extent of histological differentiation [16, 18], etc. While in clinical practice, these factors present some limitations. For example, it is hard to differentiate vascular invasion from an artefact (e.g. tissue shrinkage due to improper preparation), requiring assistance by immunohistochemistry [19]. In addition, the extent of histological differentiation is weakly reproducible among observers, which leads to heterogeneous constituent ratios (a too high ratio of moderate differentiation) [20]. In this context, looking for more morphological parameters to judge and predict LN metastasis is important and has clinical significance [21].

In recent years, TB and PDC are increasingly being recognized and considered to be potentially related to the invasive capacity of tumor cells [7, 10, 22]. Both TB and PDC have been included in The World Health Organization (WHO) Classification of Tumours of the Digestive System 2019 for reporting CRC [15], where high-grade TB may predict poor prognosis while PDC is described as a morphological feature indicating easy progression. Many studies reported the relationship between the TB/PDC in primary lesion and clinical pathological parameters (e.g., tumor size, histologic grade, depth of tumor invasion, vascular invasion, and neural invasion) [9, 23], and the results are "generalizable". However, little has been reported concerning the role of ITF pathological parameters in LN metastasis in CRC. Besides, the current studies are mostly qualitative studies on whether there is LN metastasis, without involving the number of metastasis (quantitative study). Moreover, the TB and PDC in primary tumors and LN metastases have rarely been reported simultaneously.

TB has been extensively studied for its difference between different histologic grades and potential as an independent risk factor for stage II CRC [22]. TB was added as a potential tumour-related prognostic factor in the WHO Classification of Tumours of the Digestive System’s 5th edition in 2019 [15]. The prognostic relevance of TB is reflected in publications by the Chinese Society of Clinical Oncology (CSCO) [24], as well as its inclusion in the guidelines for T1 CRC of Japan[16]. CSCO guidelines have listed TB2/3 as a high risk feature of T1 CRC [24]. However, TB is not currently included as a high risk adverse feature in several guidelines, including The National Institute for Health and Clinical Excellence (NICE) guidelines [25]. The present study found that TB may perform well in risk stratification in LN metastasis in CRC patients at other stages. Microscopically, TB can present as a single cell or a small cell cluster. In clinical practice, difficulty may exist among pathologists in identifying TB, especially in differentiating tumor cells from histiocytes and fibroblasts without assistance by immunohistochemistry [26]. In this context, PDC, another morphological parameter that can predict LN metastasis after endoscopic resection of pT1 CRC tumor, can act synergistically [12]. In a literature review that gives a summary of 12 studies [27], PDC grading was superior to the conventional histologic grading in predicting unfavorable outcomes. Our previous data based on 101 cases revealed that the PDC in primary CRC lesion had positive associations with histologic grade, depth of invasion, LN metastasis, vascular invasion, distant metastasis and TB, and it is speculated that PDC may be closely related to the invasive capability of CRC cells. Based on the findings above, we believe that the novel morphological parameters: TB and PDC, are related to the capability of CRC cells in LN metastasis, and they have the potential to be indicators used in risk stratification in LN metastasis in clinical practice. Notably, more attention is needed when high-grade TB and PDC are present in the primary lesion. It would be of great clinical significance if the conclusion can be applied to endoscopic biopsy specimens, while related further in-depth studies are warranted. On the other hand, we also realize that given the nature of single-center retrospective studies, multi-center prospective studies are more valuable. If clinical research and basic research in this field can be carried out in the future, there will be clearer application value.

## Conclusion

In this retrospective study, we evaluated both lesions of primary and LN metastases of colorectal cancer, using the TB and PDC grading systems. The TB and PDC in the invasive front of primary tumors presented significant correlations with the incidence of LN metastasis and the number of LN metastases in CRC. TB and PDC can be employed as reference indicators to assess or predict the potential of LN metastasis in CRC in clinical practice.

## Data Availability

The data is authentic and reliable. The datasets used and/or analysed during the current study are available from the corresponding author on reasonable request.

## Abbreviations

CRC: Colorectal cancer
LN: Lymph node
TB: Tumor budding
PDC: Poorly-differentiated clusters
GLOBOCAN: Global Cancer Observatory
GI: Gastrointestinal cancer
TNM: Tumor-Nodule-Metastasis
ITF: Invasive tumor front
N-: Lymph node metastasis negative
N +: Lymph node metastasis positive
AJCC: American Joint Committee on Cancer
HE: Hematoxylin-eosin
OR: Odds ratio
WHO: World Health Organization
CSCO: Chinese Society of Clinical Oncology
NICE: The National Institute for Health and Clinical Excellence
